# Development of Sustainable, Low-Shrinkage Concrete Through Optimized Aggregate Gradation, Cement Reduction, and Internal Curing

**DOI:** 10.3390/ma18102194

**Published:** 2025-05-09

**Authors:** Erfan Najaf, Maedeh Orouji, Linfei Li, Eric N. Landis

**Affiliations:** 1 Department of Civil and Environmental Engineering, University of Maine, Orono, ME 04469, USA; erfan.najaf@maine.edu (E.N.); maedeh.orouji@maine.edu (M.O.); landis@maine.edu (E.N.L.); 2 Department of Civil and Environmental Engineering, Florida International University, Miami, FL 33174, USA

**Keywords:** aggregate grading, cement reduction, internal curing, sustainability, Tarantula curve

## Abstract

The durability of concrete is compromised by early-age cracking, which provides a pathway for harmful ions and water to penetrate the material. Early-age cracking, however, is most commonly caused by concrete shrinkage. This study investigates strategies for minimizing the shrinkage of concrete by optimizing aggregate gradation via the Tarantula Curve, reducing cement content, and incorporating lightweight fine aggregates (LWFA) as internal curing agents. The commercially adopted mix design was used as a reference, with the cementitious materials-to-aggregate (C/A) ratio reduced from 0.21 (reference) to 0.15 (proposed), incorporating 0–15% LWFA replacement levels. Workability (ASTM C143), mechanical performance (ASTM C39, ASTM C78), durability (AASHTO TP 119-21), and dimensional stability (ASTM C157) were evaluated through ASTM standard tests. The results highlight that optimizing the C/A ratio cannot only improve both compressive and flexural strengths in regular concrete but also mitigate the total shrinkage by 12.68%. The introduction of LWFA further reduced shrinkage, achieving a 19.72% shrinkage reduction compared to regular concrete. In addition, the sustainability of the developed mix designs is enhanced by the reduced cement usage. A Life Cycle Assessment (LCA) based on the TRACI method confirmed the sustainability advantages of cement reduction. The optimized mix designs resulted in a 30% decrease in CO_2_ emissions, emphasizing the role of mix design in developing environmentally responsible concrete. Overall, lowering the cement amount and the addition of LWFA provide an optimal combination of shrinkage control, strength retention, and sustainability for applications.

## 1. Introduction

Shrinkage-induced cracking is one of the primary challenges compromising the durability of concrete, often leading to visible cracks within hours of placement [1]. Shrinkage, defined as the reduction in volume that occurs as concrete hardens and dries, is widely recognized as a critical factor contributing to early-age cracking and reduced infrastructure longevity. Among the various types of shrinkage, drying shrinkage and autogenous shrinkage have garnered the most attention in recent decades, as both predominantly develop during the early stages of hydration, typically within the first 28 days [2]. The severity of each type is largely influenced by the water-to-cement (w/c) ratio. When the w/c ratio exceeds 0.4, drying shrinkage tends to dominate due to excess water evaporating without reacting with cement. In contrast, autogenous shrinkage becomes more significant in high-strength concretes, where w/c ratios are typically below 0.4, due to self-desiccation within the cementitious pore structure [3]. Effectively addressing both types of shrinkage is essential for enhancing concrete durability and prolonging the service life of infrastructure [4,5].

In concrete, cementitious materials are primarily responsible for shrinkage [5]. Compared to aggregates, cementitious materials undergo significantly greater volume changes during hydration. A high cement content often results in unstable mixes, increased chemical, drying, and autogenous shrinkage, elevated heat of hydration, and a greater likelihood of cracking [6]. Well-graded aggregates can contribute to minimizing voids and reducing cement paste demand. Previous research showed that increasing aggregate volume in concrete mixtures effectively reduces shrinkage, reduces production costs, and decreases carbon dioxide emissions [7]. This reduction occurs due to two main mechanisms: first, by diluting the shrinkage potential within the cement paste matrix, and second, by providing a rigid skeletal structure that resists overall deformation and enhances the mechanical performance [4]. Guimarães’ work indicates that refining the granular skeleton through optimized grading improves packing density and structural compactness, leading to better mechanical properties regardless of aggregate shape, size, or composition [8]. A similar conclusion was made by Guan et al. that structuring aggregate particle sizes to achieve optimal gradation and compactness is recognized as a beneficial approach to improving concrete properties [9]. Ghoddousi et al. investigated that the packing density of aggregates and cement content play critical roles in not only shrinkage mitigation, but also influence the strength, durability, elastic modulus, and creep behavior [10]. Qin et al. applied Fuller’s grading curve in numerical simulations and demonstrated its effectiveness in optimizing concrete compactness and strength [11]. A study carried out by Ukala highlighted intercorrelation between flowability and shrinkage, suggesting that optimal aggregate size selection can mitigate shrinkage without sacrificing workability [12]. Additionally, aggregate composition matters: concrete made with mined aggregates tends to exhibit lower shrinkage than that made with crushed aggregates [13]. From the above literature, increasing the aggregate volume and optimizing its gradation are expected to reduce total cement content in the mixture, thereby mitigating shrinkage. This approach also contributes to sustainability by lowering the carbon footprint of concrete. Cement production is a major contributor to global carbon emissions, making its reduction a key goal in sustainable construction [14].

Internal curing is another effective method for shrinkage mitigation, particularly autogenous shrinkage. This technique involves the incorporation of internal water reservoirs, such as pre-wetted lightweight aggregates (LWA) and superabsorbent polymers, into the concrete mixture to maintain internal moisture levels during hydration [15]. By promoting internal humidity through gradual water release, internal curing supports sustained cement hydration, reducing self-desiccation and enhancing performance [16,17,18]. Internal curing is especially valuable in high-performance concrete (HPC), where it significantly reduces early-age autogenous shrinkage and cracking [19,20]. Studies by Zhang et al. and Chen et al. showed that pre-soaked lightweight aggregates (PS-LWA) not only help control shrinkage and maintain internal humidity but also enhance compressive strength [17,21,22]. A similar conclusion was made by Chatale that LWA serves as an internal curing agent that can effectively mitigate autogenous shrinkage and cracking. However, the overall strength reduction was observed [23]. A practical implementation was conducted by Lafikes et al. by combining the pre-wetted fine LWA as the internal curing agent with supplementary cementitious materials (SCMs) for bridge deck casting [24]. As a result, a more durable infrastructure was achieved. Alternative internal curing agents, such as crushed clay bricks or recycled aggregates, have also demonstrated effectiveness in mitigating autogenous shrinkage [25].

Despite extensive research on cement reduction, aggregate optimization, and internal curing, a significant gap remains in developing a synergistic approach that simultaneously addresses shrinkage mitigation, mechanical performance, and environmental sustainability. This gap is especially critical in cold and coastal regions, where freeze-thaw damage and chloride-induced corrosion pose major durability challenges. Early-age cracking—primarily caused by shrinkage—can create pathways that allow harmful ions (e.g., chloride, sulfide) and moisture to rapidly penetrate the concrete, severely compromising its long-term durability. This study addresses this gap and provides a comprehensive analysis of the synergistic effect by combining Tarantula curve-based aggregate optimization with internal curing using lightweight fine aggregates (LWFA). The novelty of this research lies in its comprehensive, climate-resilient approach that merges three strategies—Tarantula gradation, internal curing using LWFA, and Life Cycle Assessment (LCA) via TRACI—to achieve both structural performance and environmental benefits. The Tarantula curve provides a practical framework to optimize aggregate proportions, improve workability, and reduce cement paste content. This method is widely accepted by state Departments of Transportation (DOTs) across the U.S., with practical relevance in the field [26,27,28]. By promoting efficient aggregate packing, the Tarantula curve minimizes voids and improves performance. The proposed mix design incorporates both Tarantula curve optimization and LWFA-based internal curing to reduce cement content, limit environmental impact, and mitigate shrinkage while maintaining structural integrity. The inclusion of LWFA not only supports internal curing but also sustains workability and durability. To evaluate the proposed approach, a systematic experimental program was conducted, including tests for workability, shrinkage, compressive and flexural strength, and bulk resistivity across varied C/A ratios and LWFA contents. The results were further evaluated through LCA to quantify the environmental advantages of the proposed mix designs. The following sections outline the experimental program and key findings. Section 2 describes the materials and experimental methods; Section 3, Section 4 and Section 5 present and discuss the results; Section 6 evaluates the sustainability of the proposed mixtures through LCA; and Section 7 summarizes the main conclusions and practical implications.

## 2. Experimental Program

### 2.1. Materials

Ordinary Portland Cement-Type II, Type 1L (PLC), and Grade 120 Slag are the cementitious materials adopted in this study, provided by Dragon Cement, ME, USA. The chemical compositions of Type II cement, PLC, and slag chemical characteristics are listed in Table 1, which complies with the ASTM C114-18 standard.

To meet the workability requirement, the polycarboxylate-ether-based high-range water reducer (HRWR) provided by BASF, MA, USA, was selected and used based on polycarboxylate chemistry in accordance with ASTM C1017 and ASTM C494 standards. HRWR was necessary to maintain sufficient workability, especially in mixes with reduced paste content (lower C/A ratios), where flowability would otherwise be compromised.

**Figure 1:**
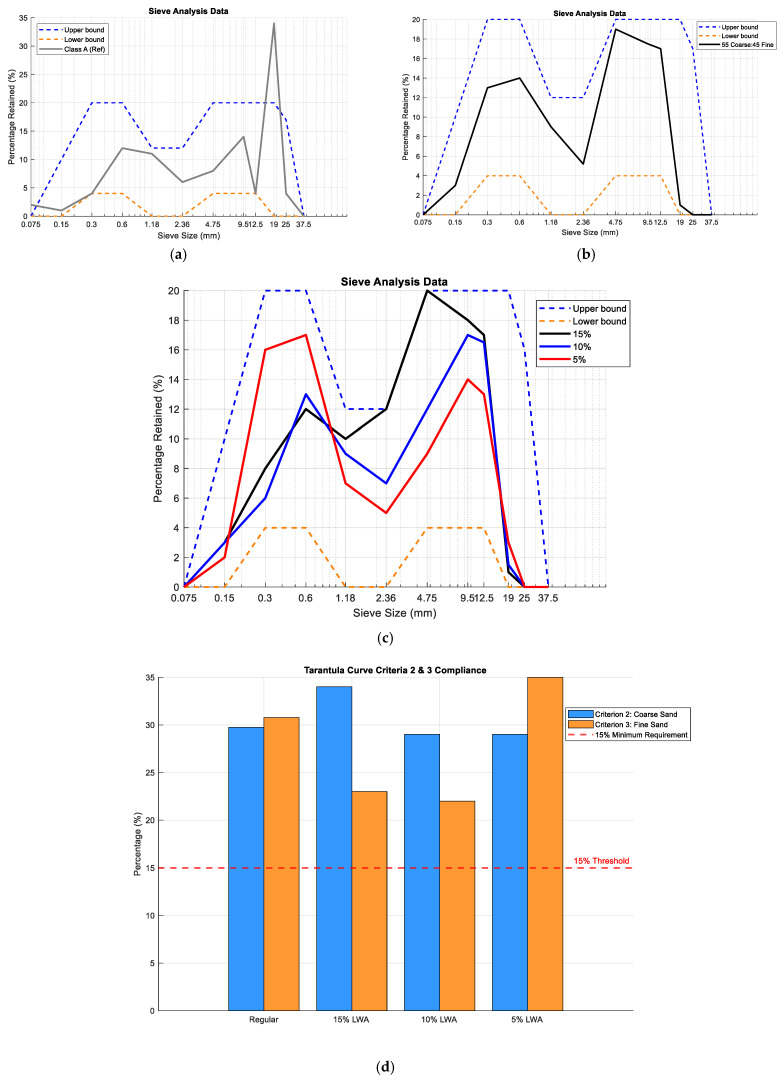
Aggregate grading based on the Tarantula curve. (**a**) Maine DOT mix design. (**b**) Optimized mix using the Tarantula curve. (**c**) Gradation curves for LWFA introduction. (**d**) Compliance of regular and LWFA-modified mixes with Criterion 2 and Criterion 3.

The optimization of the aggregate ratio within concrete mixes was critically examined to adhere to the Tarantula curve specifications, as illustrated in Figure 1a–d. The study applied two types of aggregates: a regular aggregate mix and the incorporation of LWFA from Norlite Agg, NY, USA, both of which were evaluated for compliance with the Tarantula curve. The Tarantula curve provided upper and lower bounds, depicted in blue and orange, respectively, which are essential for determining the optimal aggregate gradation for improved concrete quality and workability. According to the definition of the Tarantula curve, three criteria were applied (Table 2): (1) aggregate gradation should fall within the upper and lower bounds of the Tarantula curve. This ensures that the particle size distribution is well-proportioned to provide good workability, durability, and strength; (2) at least 15% of the total aggregate volume should consist of coarse sand particles, defined as particles passing the #8 sieve (2.36 mm) but retained on the #30 sieve (0.6 mm). This fraction is crucial for enhancing workability by providing proper interlocking and particle stability; and (3) at least 15% of the total aggregate volume should consist of fine sand particles, defined as particles passing the #30 sieve (0.6 mm) but retained on the #200 sieve (0.075 mm). These particles help fill voids and contribute to the paste structure, preventing excessive bleeding or segregation.

The existing Class A concrete mix used by Maine DOT is indicated in gray in Figure 1a. The ‘Class A’ mix refers to the standard concrete mix currently used by the Maine DOT for general-purpose construction, with a coarse-to-fine aggregate ratio of 68:32. This mix does not meet the Tarantula curve requirements, as it falls outside the upper and lower bounds (Criterion 1). This suggests that the mix may have suboptimal performance in terms of workability and strength distribution. To address this issue, the aggregate ratio was adjusted to 55:45, as depicted in Figure 1b, ensuring compliance with ASTM C33M-18 standards for high-quality concrete. The water absorption rates for regular fine aggregate and coarse aggregate were measured as 1.24% and 0.94%, respectively. Figure 1b confirms that the adjusted 55:45 aggregate ratio meets Criterion 1, staying within the defined gradation limits. Figure 1c presents the gradation curves for the LWFA-modified concrete mixes where 0–15% of the regular fine aggregate was replaced by LWFA, confirming that the introduction of LWFA up to 15% still falls within the Tarantula curve limits, meeting Criterion 1. Figure 1d separately displays the Criterion 2 and 3 evaluations for both regular concrete (55% Coarse–45% Fine) and LWFA-modified mixes. The results confirm that optimized mixes satisfy both Criterion 2 and Criterion 3.

### 2.2. Sample Preparation

This research started with six regular mixing designs without LWFA. The first mix utilized Type II cement, while PLC was used in the remaining five mixes. The study primarily aimed to evaluate the effects of reducing the cement content by adjusting the C/A ratio but also compared the performance between Type II cement and PLC. The first and second mix designs were established as control samples to provide a baseline for comparison. The incorporation of LWFA is provided from mix designs 7 to 18 with various replacement ratios, as shown in Table 3.

In preparing the concrete, all ingredients–cement, aggregates, water, and admixtures–were measured and introduced into the drum mixer in a consistent sequence. For mixtures containing LWFAs, the LWFA was pre-soaked in water at room temperature for a minimum of 24 h to allow full absorption. Immediately before mixing, the LWFA was drained to a saturated surface-dry (SSD) condition to avoid unintended water loss or gain in the concrete mix. This step ensured that the LWFA could release internal moisture gradually for internal curing. Once the aggregates and cement were placed in the mixer, water and high-range water reducers (HRWR) were added as needed to achieve the desired slump. The mixer was operated for several minutes until the mixture achieved a uniform consistency. Because of the reduced paste content in some mixes, additional HRWR was required to maintain the target workability. To control the consistency, identical samples were prepared for each test and each mix design. All the samples from each mix design were batched at one time. After casting, the specimens were cured in the environmentally controlled room for 24 h before demolding at 23 ± 2 °C and 95% RH. After demolding, all the samples were continuously cured in the wet room except the shrinkage samples, which were left in the air exposure to enlarge the shrinkage effect.

### 2.3. Experimental Plan

Four different standardized testing methods were adopted to evaluate the performance of developed concrete mix designs: slump, compressive strength, flexural strength, bulk resistivity, and total shrinkage. The slump test, followed by ASTM C143, was applied to evaluate the workability of concrete before casting specimens in molds. The compression test followed ASTM C39 with adaptations specific to our mixtures. Cylindrical specimens (100 × 200 mm) were cast for each mix. The cylinder specimens were tested at 7, 14, and 28 days. A load-control method was used, applying load at 0.25 MPa/s until failure. The maximum load was recorded, and compressive strength was calculated from the specimen’s cross-sectional area. Flexural strength was measured at 28 days only, as per ASTM C78, since this value is most relevant for service life assessment, and early-age values are less indicative of long-term bending performance in shrinkage-sensitive applications. Beam specimens (150 × 150 × 500 mm) were tested at 28 days. A three-point loading configuration was employed, and the load was applied at a controlled rate of 0.25 mm/min specified in ASTM C78. Specimens were loaded until a clear fracture developed, and the maximum load at rupture was recorded. Flexural strength/modulus of rupture was computed from the beam dimensions and the fracture load. Bulk resistivity measurements were performed to assess the concrete’s potential durability and resistance to chloride penetration. Additional cylinders (100 × 200 mm) were tested at 28 and 56 days. The specimens were presoaked in water to reach SSD condition. The specimens were placed in a specialized cell that applied a small AC voltage across the specimen ends. The resulting current was measured, and resistivity was calculated based on specimen geometry, voltage, and current. Higher resistivity values typically indicate a denser microstructure and hence a lower permeability. Each data point represents the mean of three specimens. Shrinkage was measured per ASTM C157 but with specific adaptations to capture both early-age and longer-term drying shrinkage. Prismatic specimens with dimensions of 76 × 76 × 286 mm were cast, covered to prevent moisture loss, and kept in molds for ~24 h in the curing room. Right after demolding, the initial comparator reading was recorded as the reference length. Specimens were then exposed to air with 23 ± 2 °C and ~50% R.H. to encourage drying. This was done deliberately to capture both autogenous and drying shrinkage starting from an early age. Length changes were measured daily up to 21 days. We discontinued measurement once the incremental shrinkage changes became negligible. Equation (1) was used to calculate the percentage length change:

(1) $$\%\;Length\;Change=\frac{Length\;at\;any\;time-Initial\;Length}{Gauge\;Length\times 100}$$

The experimental program follows a two-step approach. In the first step, cement reduction is achieved by optimizing aggregate size gradation using the Tarantula curve. As expected, improved packing will enhance mechanical performance, resistivity, and dimensional stability. In the second step, LWFA will be introduced to further reduce total shrinkage, with a specific focus on mitigating autogenous shrinkage. Mechanical performance and resistivity will be tested and evaluated to assess the overall effectiveness of the approach. Each test was conducted on three specimens to ensure repeatability, and the results were reported as mean values with standard deviation where appropriate. Table 4 shows the summary of tests performed.

## 3. Testing Results for Regular Concrete

### 3.1. Slump Test

The slump testing results are provided in Table 5. The reference mix design 1 with Type II cement had a slump of 132 mm, while the reference mix design 2 with PLC exhibited a slightly higher slump of 139 mm, likely due to the smoother morphology of limestone powder compared to cement. Mix designs 3 and 4 showed slumps of 127 mm and 114 mm, respectively, indicating that a lower C/A ratio reduces workability, as less paste is available to lubricate aggregate particles. Mix design 5 had a significantly low slump of 73 mm, suggesting a stiff mix that may be difficult to place and finish. However, mix design 6, while still on the lower end of workability, improved to a slump of 89 mm compared to the 0.15a (PLC) sample. This improvement resulted from adjusting the coarse-to-fine aggregate ratio from 55-45 to a more balanced 50-50, enhancing particle distribution and slightly improving workability.

### 3.2. Mechanical Performance

**Figure 2:**
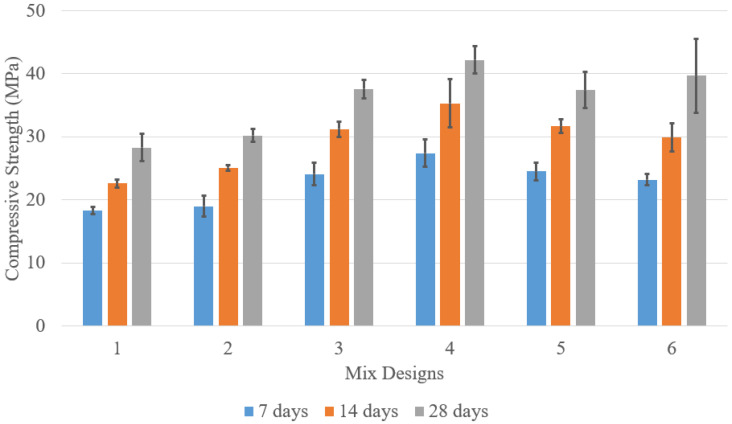
Comparison between compressive strengths.

The compressive strength of concrete mix designs 1–6 is shown in Figure 2. As expected, strength increases over time with curing. The data includes specimens with varying C/A ratios and two types of cement: Type II and PLC. For C/A = 0.21, mix design 1 consistently achieved higher compressive strength than mix design 2 at 7, 14, and 28 days, likely due to PLC’s finer particles, which promote a denser microstructure and enhanced hydration. As the C/A ratio decreased, compressive strength increased, with mix design 4 (C/A = 0.165) reaching the highest 28-day strength of 42.2 MPa. This improvement is attributed to better particle packing and interlocking, optimizing the paste-to-aggregate balance. However, mix design 5 showed lower strength than mix design 4 due to poor packing caused by low workability and segregation.

**Figure 3:**
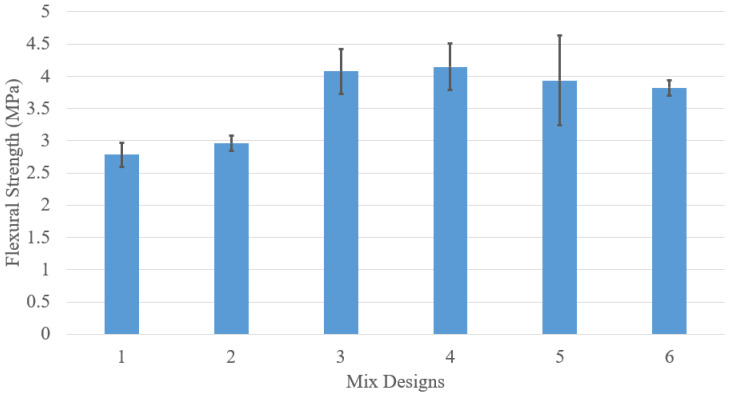
Comparison between flexural strength after 28 days of curing.

The analysis of flexural strength across concrete specimens with varying C/A ratios and cement types, shown in Figure 3, reveals a clear trend in bending resistance. The reference mix design 1 exhibited a flexural strength of 2.78 MPa. When PLC replaced Type II cement at the same C/A ratio, strength increased to 2.95 MPa, a 6.19% improvement, due to PLC’s denser microstructure and enhanced bonding. As the C/A ratio decreased to 0.18 and 0.165, flexural strength rose significantly to 4.08 MPa and 4.14 MPa, representing 46.29% and 48.76% improvements over the baseline. This suggests reduced paste ratios enhance strength through better aggregate packing and minimized micro-cracking. The highest flexural strength occurred in mix design 4, indicating an optimal P/A balance. However, further reducing the paste ratio to 0.15 in mixes 5 and 6 led to lower flexural strengths of 3.94 MPa and 3.82 MPa, respectively. Despite adjusting the aggregate ratio from 55-45 to 50-50, these mixes saw strength reductions of 5.00% and 7.82% compared to mix design 4 due to insufficient paste for effective aggregate bonding, making the mix less resilient under bending stress. Our results are consistent with previous studies showing that optimized particle packing enhances strength. For instance, Ghoddousi et al. [10] and Qin et al. [11] demonstrated that denser aggregate packing improves compressive strength and reduces microcracking. Similarly, Ťažký et al. [13] found that composition significantly affects volume stability and strength development.

### 3.3. Bulk Resistivity

**Figure 4:**
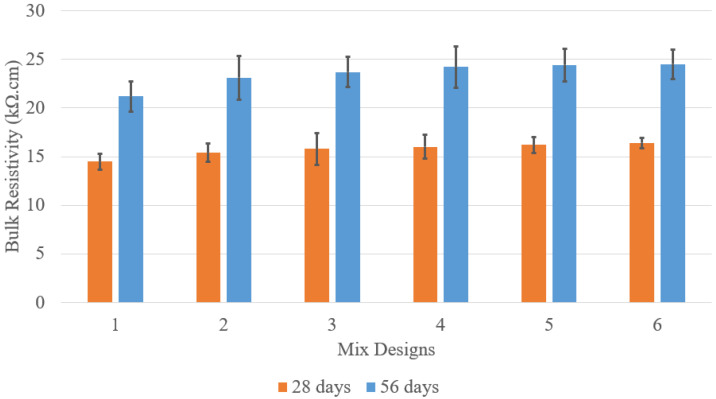
Bulk resistivity results.

As shown in Figure 4, bulk resistivity is a key indicator of concrete’s ability to resist electrical current flow, indirectly reflecting its permeability and durability against chloride-induced corrosion. The data presents bulk resistivity measurements for concrete specimens with varying C/A ratios at 28 and 56 days. Mix design 1, the baseline, recorded resistivity values of 14.5 kΩ·cm at 28 days and 21.2 kΩ·cm at 56 days, representing conventional concrete’s inherent resistance. Mix design 2 showed a slight increase to 15.4 kΩ·cm at 28 days and 23.1 kΩ·cm at 56 days due to PLC’s finer particle distribution and secondary hydration, which refines the pore structure and reduces conductivity. As the C/A ratio decreased, bulk resistivity progressively increased. Mix designs 3–6 followed an ascending trend, reaching 16.4 kΩ·cm at 28 days and 24.5 kΩ·cm at 56 days. This suggests that a denser, less porous aggregate structure enhances electrical resistance. The consistent improvement in 56-day measurements over 28-day results indicates ongoing hydration and microstructure refinement. This trend highlights that optimizing the C/A ratio can improve resistance to ionic transport, potentially extending the service life of concrete structures in aggressive environments.

### 3.4. Shrinkage

**Figure 5:**
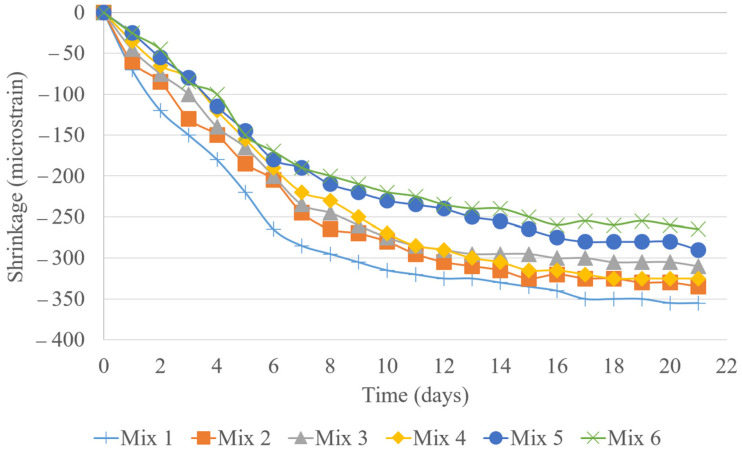
Shrinkage test results.

The shrinkage data over a 22-day period highlights the influence of the cement type and C/A ratio on concrete shrinkage. Mix design 1, the baseline, exhibited the highest shrinkage at 355 με. In contrast, mix design 2 showed improved performance with 335 με, a 5.63% reduction, due to PLC’s denser microstructure. Further reductions were observed as the C/A ratio decreased, with mix designs 3 and 4 (0.18 and 0.165 C/A) reaching 310 and 325 με, respectively, up to a 12.68% decrease from the Type II baseline. Mix designs 5 and 6 continued this trend, registering 285 and 265 με, marking a reduction of up to 19.72%. This suggests that lowering the paste content effectively minimizes shrinkage. The lowest shrinkage in mix design 6 underscores the role of an optimal P/A balance in mitigating shrinkage-related issues. As illustrated in Figure 5, reducing the C/A ratio can enhance dimensional stability, offering a practical strategy for designing more durable concrete with reduced cracking and maintenance needs, ultimately improving the longevity of concrete structures.

## 4. Testing Results for LWFA Concrete

### 4.1. Mechanical Performance

**Figure 6:**
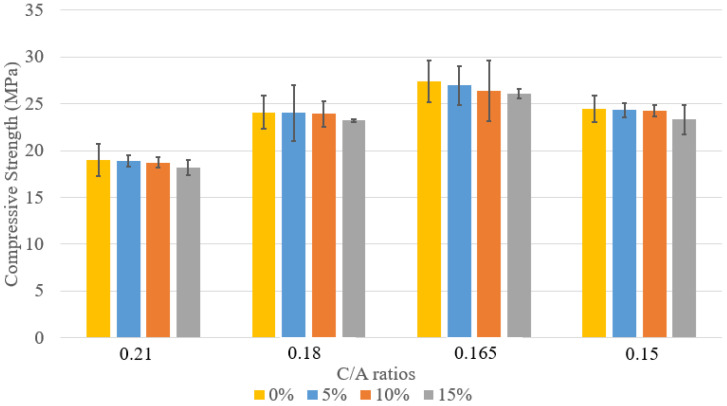
Compressive strength at 7 days of curing with various levels of LWFA introduction.

**Figure 7:**
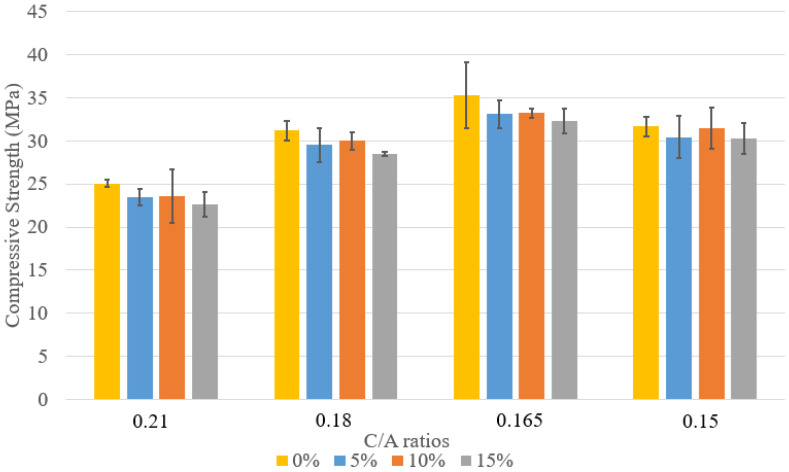
Compressive strength at 14 days of curing with various levels of LWFA introduction.

**Figure 8:**
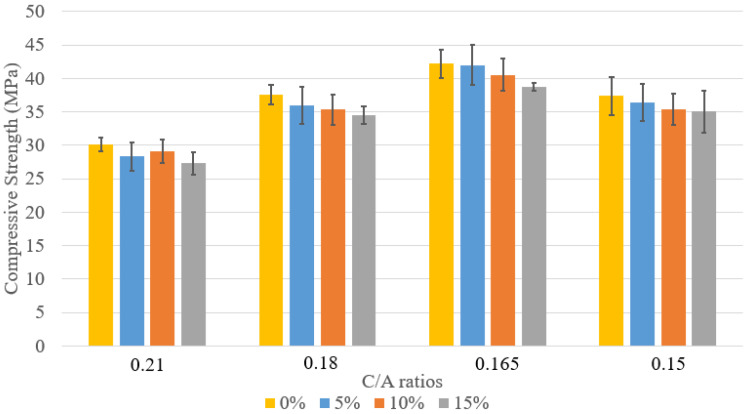
Compressive strength at 28 days of curing with various levels of LWFA introduction.

The comparative analysis (Figure 6, Figure 7 and Figure 8) of compressive strength across concrete mixes with 5%, 10%, and 15% lightweight aggregate (LWA) under varying cement-to-aggregate (C/A) ratios highlights key insights. In the 5% LWA group, the 0.165 PLC mix achieved the highest 28-day strength of 42.0 MPa, significantly outperforming the 0.21 PLC mix (28.4 MPa). This improvement reflects the benefits of optimized aggregate packing and reduced cement paste content, rather than increased cement usage.

As LWA content increased to 10% and 15%, compressive strength declined slightly, indicating that higher LWA levels can compromise strength, especially when paste volume becomes insufficient to maintain proper bonding.

**Figure 9:**
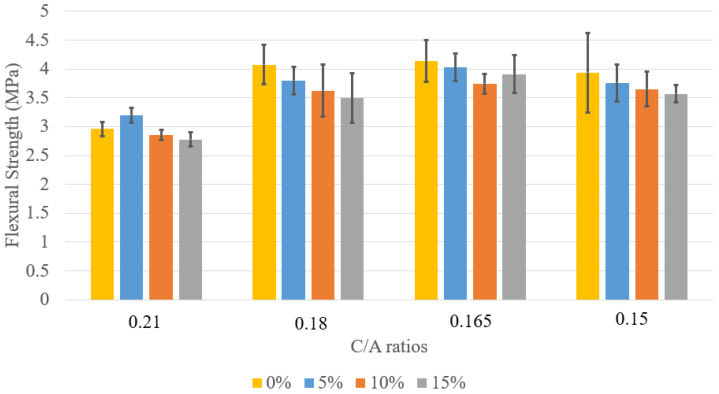
Flexural strength at 28 days for LWFA concrete.

As shown in Figure 9, for C/A = 0.21, increasing LWFA content from 5% to 15% reduces flexural strength from 3.20 MPa to 2.78 MPa, a 13.1% decline, mirroring the trend observed in compressive strength. This suggests that higher LWFA content at this C/A ratio negatively impacts flexural strength. For C/A = 0.18, flexural strength peaked at 5% LWFA (3.80 MPa) but slightly declined at 10% (3.63 MPa) and 15% (3.50 MPa), indicating that exceeding 5% LWFA may not be beneficial. In contrast, the C/A = 0.165 mix showed a steady increase in flexural strength with higher LWFA, reaching 3.91 MPa at 15%, suggesting improved structural performance. For C/A = 0.15, flexural strength varied, peaking at 3.76 MPa with 5% LWFA but decreasing at higher LWFA levels (3.65 MPa at 10%, 3.57 MPa at 15%). This suggests a complex interaction between LWFA content and paste composition. Overall, the C/A = 0.165 mix achieved the highest flexural strengths across all LWFA levels, demonstrating its effectiveness in balancing cement content and LWFA for optimal performance. The C/A = 0.18 mix also performed well at lower LWFA levels, offering a viable balance between structural strength and cement reduction.

### 4.2. Bulk Resistivity

**Figure 10:**
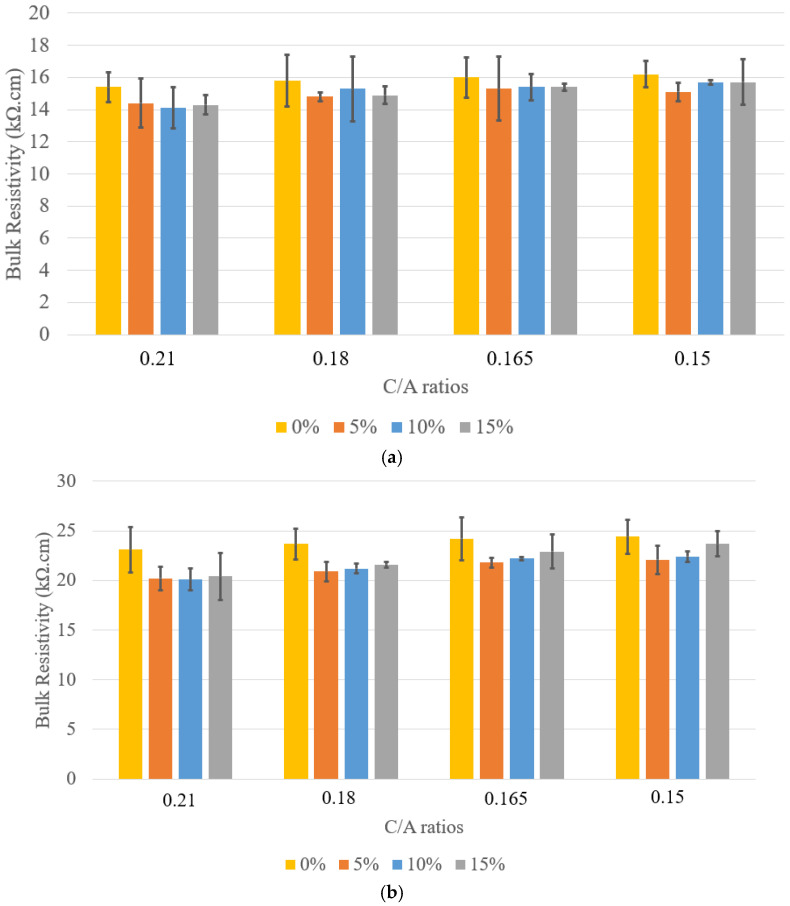
Bulk resistivity for LWFA concrete at (**a**) 28 days and (**b**) 56 days.

The bulk resistivity results for concrete specimens with 5%, 10%, and 15% LWFA across various C/A ratios are presented in Figure 10. At 5% LWFA, the C/A = 0.15 mix exhibited the highest bulk resistivity at 56 days (22.1 ± 2 kΩ·cm), indicating that a lower C/A ratio (higher cement content) enhances resistivity within this LWFA category. Conversely, the C/A = 0.21 mix, with the highest C/A ratio, recorded the lowest resistivity (20.2 ± 2 kΩ·cm), suggesting that reduced cement content improves resistivity in concrete with lower LWFA percentages. For 10% LWFA specimens, the trend persists, with the C/A = 0.15 mix again showing the highest resistivity at 56 days (22.4 ± 2 kΩ·cm). However, the resistivity differences between mixes narrow slightly, indicating that increased LWFA content begins to significantly influence concrete’s electrical properties.

A notable shift occurs in the 15% LWFA specimens. The C/A = 0.15 mix still demonstrates the highest resistivity (23.7 ± 2 kΩ·cm at 56 days), but the increase compared to lower LWFA percentages is more pronounced. This suggests that at higher LWFA levels, the combined effect of higher cement content and increased LWFA leads to a substantial improvement in resistivity. Overall, while increasing LWFA content generally enhances bulk resistivity, the C/A ratio remains the dominant factor. Higher cement content consistently results in lower resistivity, regardless of LWFA percentage, likely due to improved particle packing, which restricts electrical current flow. These findings highlight the importance of optimizing cement-to-aggregate ratios to achieve superior electrical properties, particularly in applications requiring enhanced durability and resistance to environmental factors such as corrosion.

### 4.3. Shrinkage

**Figure 11:**
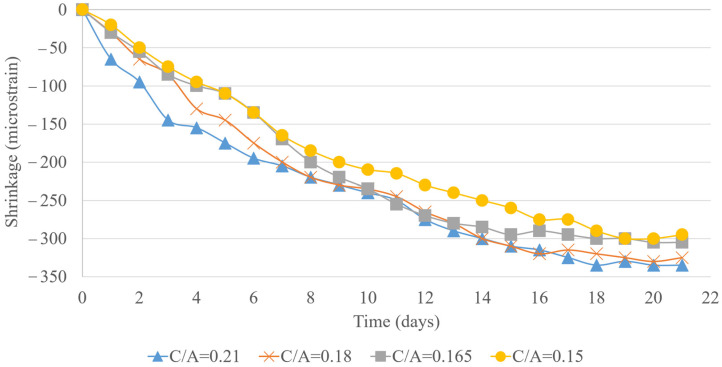
Total shrinkage for 5% lightweight replacement.

**Figure 12:**
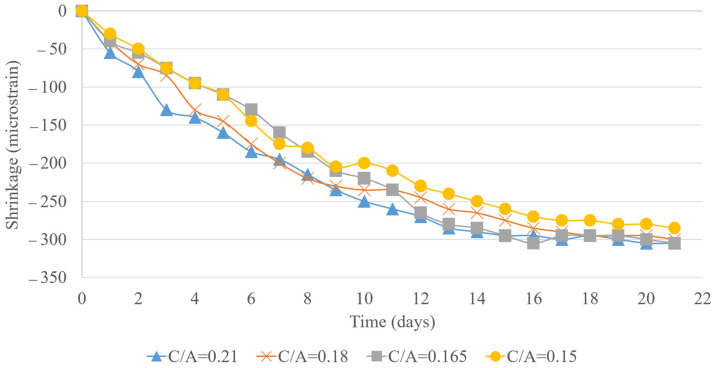
Total shrinkage for 10% lightweight replacement.

**Figure 13:**
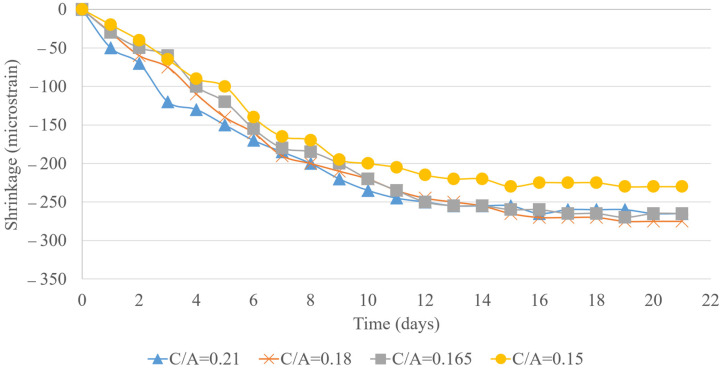
Total shrinkage for 15% lightweight replacement.

The analysis of 22-day shrinkage measurements for concrete samples with 5%, 10%, and 15% LWFA replacement across various C/A ratios provides valuable insights into the effectiveness of LWFA in mitigating shrinkage and the influence of C/A ratios on this behavior, as shown in Figure 11, Figure 12 and Figure 13. At 5% LWFA replacement, mixes with C/A ratios of 0.15 and 0.165 exhibited similar levels of shrinkage mitigation, suggesting that at lower LWFA levels, the specific C/A ratio has a minimal effect on shrinkage as long as the cement content remains relatively high. With 10% LWFA replacement, the mix with a C/A ratio of 0.15 demonstrated the most effective shrinkage reduction, while no significant difference was observed between the 0.165 and 0.18 C/A ratio mixes. This indicates that increasing LWFA replacement to 10% enhances shrinkage mitigation, particularly in mixes with higher cement content. At 15% LWFA replacement, the trend continued, with the 0.15 C/A ratio mix showing the best shrinkage reduction. Again, mixes with C/A ratios of 0.165 and 0.18 displayed similar shrinkage levels, reinforcing the role of higher cement content in effectively reducing shrinkage, even as LWFA content increases. The incorporation of LWFA positively influences shrinkage mitigation across all mix designs, with higher LWFA percentages generally leading to further reductions in total shrinkage. When combined with a lower cement content approach, LWFA effectively minimizes both drying and autogenous shrinkage, making it a promising strategy for enhancing dimensional stability across a wide range of water-to-cement ratios. The summary of testing results for C/A = 0.15 is provided in Table 6 as an example.

## 5. Sustainability Investigation

Cement production is energy-intensive and responsible for substantial greenhouse gas emissions, particularly CO_2_. Through the comprehensive performance-based investigation above, the cement amount in the matrix can be effectively reduced. Therefore, it is worth taking a look at the sustainability of new concrete mixes via LCA.

### 5.1. Methodology

**Figure 14:**
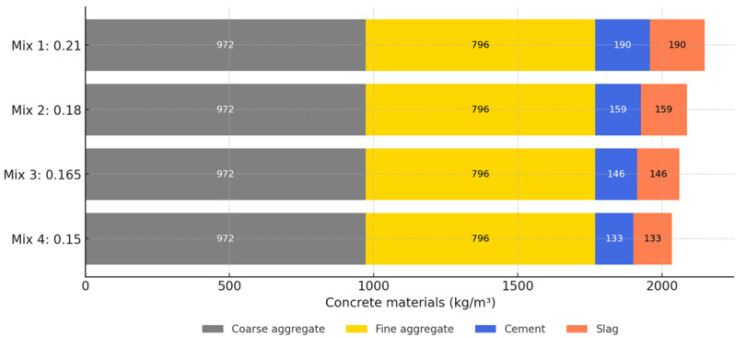
Mix designs for sustainability investigation.

**Figure 15:**
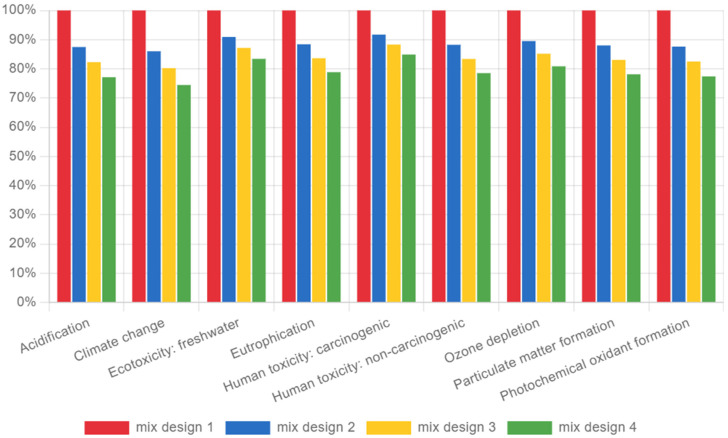
Environmental impact analysis.

The study utilized the Tool for the Reduction and Assessment of Chemical and other environmental Impacts (TRACI) version 2.1 and the Intergovernmental Panel on Climate Change (IPCC) guidelines for assessing various environmental impacts. The LCA was conducted from a cradle-to-grave perspective, encompassing raw material extraction, production, usage, and end-of-life phases [29]. This comparative assessment provided insights into the environmental impacts associated with each product system from a cradle-to-grave perspective. It also considered potential variations in mixed designs, regional differences in production methods, transportation distances, and end-of-life management practices. The functional unit for this LCA study is defined as 1 cubic meter (m^3^) of concrete. Figure 14 and Figure 15 presents four mix designs investigated in this section, which were previously discussed and analyzed for their mechanical performance and durability behavior as C/A = 0.21, 0.18, 0.165, and 0.15 without LWFA.

### 5.2. Environmental Impact Analysis

In Figure 15, the analysis covered several impact categories, including Global Warming Potential (GWP), acidification, eutrophication, ecotoxicity, and human toxicity. The results highlighted the environmental benefits of reducing the cement content in concrete mixes.

1. Global Warming Potential (GWP): Mix design 4 showed the lowest GWP, demonstrating the least CO_2_ emissions among the four designs. This reduction is attributed to the lower cement content, which directly correlates with lower emissions during production.
2. Acidification and Eutrophication: Similar trends were observed in acidification and eutrophication categories, with mix design 4 again showing the lowest impacts. The reduction in clinker content in the cement mix directly reduced sulfur dioxide (SO_2_) and nitrogen oxides (NOx) emissions, which contribute to acidification and eutrophication.
3. Ecotoxicity and Human Toxicity: Lower cement content also led to reductions in ecotoxicity and human toxicity metrics, particularly in mix designs 3 and 4. These mixes exhibited lower emissions of toxic substances, thus reducing potential harm to aquatic and terrestrial ecosystems, as well as human health risks.
4. Other Impact Categories: In categories such as ozone depletion and particulate matter formation, the differences between the mix designs were less pronounced. However, the overall trend favored mixes with lower cement content.

Based on the Life Cycle Impact Assessment (LCIA) results for the four concrete mix designs, significant variations in environmental impact indicators were observed. Key impact categories such as climate change (CO_2_ emissions) and global warming potential (GWP) were crucial metrics for evaluating the sustainability of each mix design. The LCIA revealed that mix design 4, characterized by a C/A ratio of 0.15, consistently exhibited the lowest environmental impact across all categories. Mix design 4 showed the most substantial reduction in CO_2_ emissions, approximately 30% lower than the reference mix design. This reduction is primarily attributed to the lower cement content, which directly correlates with decreased greenhouse gas emissions. The GWP for mix design 4 was significantly lower, reflecting a marked decrease in its contribution to climate change. The efficient use of materials and optimized aggregate grading played a critical role in minimizing the environmental footprint.

**Figure 16:**
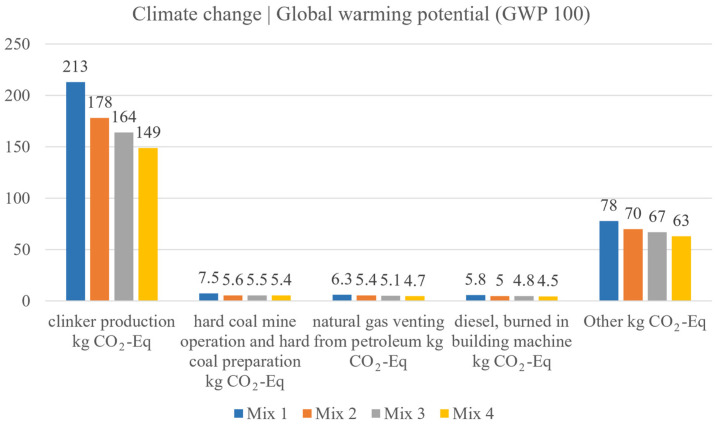
Global warming potential compared between mix designs 1–4.

Figure 16 illustrates the environmental impact of different concrete mix designs, with a focus on their contribution to global warming potential (GWP100) measured in CO_2_ equivalents. Notably, mix design 4 demonstrates the most significant reduction in CO_2_ emissions across several key categories compared to the other mixes. For instance, CO_2_ emissions from clinker production, which is the largest contributor to GWP, are reduced by 30% in mix design 4 (149 kg CO_2_-Eq) compared to mix design 1 (213 kg CO_2_-Eq). This substantial reduction underscores the environmental benefits of reducing cement content in the mix design. Similarly, other emission sources, such as diesel combustion in building machinery, show a notable decrease, with mix design 4 producing 4.51 kg CO_2_-Eq, a 22.3% reduction from mix 1 (5.8 kg CO_2_-Eq). These results highlight the effectiveness of the proposed mix design in minimizing the overall environmental footprint of concrete production, making mix 4 the most sustainable option among those tested.

**Figure 17:**
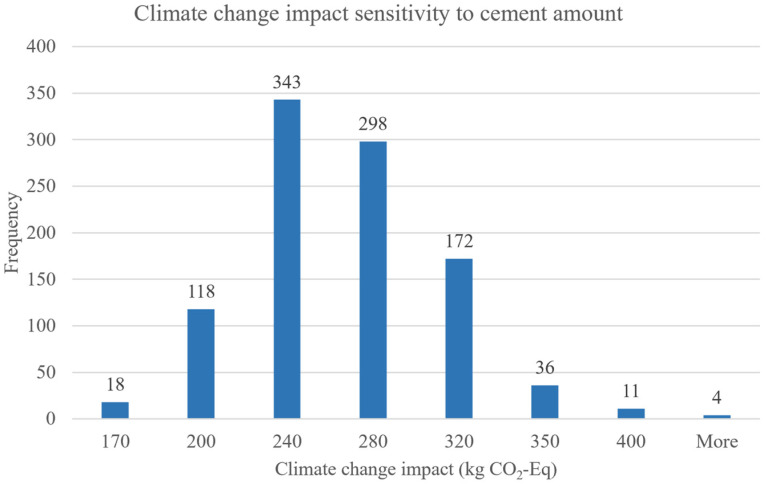
Climate change impact sensitivity to cement amount.

Figure 17 shows a range of GWP outcomes measured in kilograms of CO_2_ equivalents (kg CO_2_-Eq), segmented by different cement amounts. The bins on the *x*-axis represent different GWP values corresponding to increasing amounts of cement used in the concrete mix. The highest frequency of GWP outcomes occurs at around 240 kg and 280 kg CO_2_-Eq. This indicates that the most common GWP results from typical amounts of cement used fall within these ranges, suggesting a central tendency in the data. As the amount of cement increases to 320 kg and beyond, the frequency of occurrences gradually declines. This could imply that higher cement amounts, while contributing to higher GWP, are less frequently used or studied in typical concrete formulations. The sensitivity analysis demonstrates that the GWP of concrete is highly sensitive to the amount of cement used. Lower amounts of cement consistently result in lower GWP values, highlighting the potential for significant environmental impact reductions through material optimization and innovative mix designs that reduce cement content. The sustainability improvements observed here mirror the findings of Gursel et al. [29] and Tang et al. [30], who highlighted that cement quantity is a dominant factor in the environmental impact of concrete. Campos et al. [31] also confirmed that partial replacement of cement through optimized gradation or fillers reduces GWP significantly.

## 6. Discussion

A clear trend was observed in both regular and LWFA concretes in that, as the cement content decreased, total shrinkage consistently declined. In the concrete matrix, aggregates act as inert fillers with stable dimensions, while cement, though quasi-brittle, undergoes more significant deformation under sustained internal or external loads, leading to creep and shrinkage. Reducing the volume of cement helps minimize overall shrinkage. By incorporating the application of the Tarantula curve, the testing results show that it cannot only densify the microstructure of concrete and improve the strength but also lower cement content, which directly lowers the potential of shrinkage of concrete. However, a threshold was identified at a C/A ratio of 0.165. This is mainly due to the lack of binder in the matrix, where the aggregate needs a minimum amount of binder to control the workability of concrete. This resulted in visible defects and decreased strength, despite the benefit of lower shrinkage.

To further mitigate the cracking risks induced by shrinkage, the impact of adding LWFA was investigated. The compressive strength at 28 days gradually decreases with increasing LWFA percentages. As an example of a C/A ratio of 0.15 mix, a 5% LWFA addition results in a modest reduction of about 2.7% (36.4 MPa) compared to regular concrete (37.4 MPa), while a 15% LWFA addition shows a more pronounced decrease of approximately 6.3% (35.1 MPa). This is attributed to the porous microstructure of LWFA, where its strength and stiffness are lower than traditional solid fine aggregates. This trend suggests a trade-off between the weight reduction benefits of LWFA and the compressive strength of the concrete. A similar pattern is observed in the flexural strength test, where higher LWFA content leads to a decrease in strength. For 5% LWFA, the reduction is 4.6% (3.76 MPa), and for 15% LWFA, it is 9.3% (3.57 MPa), compared to regular concrete (3.94 MPa). These changes highlight the importance of balancing structural requirements and weight considerations in concrete design. Shrinkage analysis reveals improved performance with LWFA. The 15% LWFA mix, in particular, demonstrates a significant shrinkage reduction of about 20.7% (230 με) compared to the reference mix design (290 με). Such a significant reduction of shrinkage is attributed to the reduction of autogenous shrinkage, where LWFA serves as the internal curing agent. The bulk resistivity slightly decreases with the incorporation of LWFA, but the reduction is minimal. The 15% LWFA mix shows a 3.3% decrease in bulk resistivity (23.7 kΩ·cm) compared to regular concrete (24.5 kΩ·cm), suggesting that the electrical properties of the concrete are not significantly compromised by LWFA. The additional water carried by LWFA into the concrete matrix can potentially promote the hydration of concrete. With fewer cracking risks from shrinkage, it is reasonable to have negligible electrical resistivity reduction. However, further microstructure analysis is needed for further investigation.

From a sustainability point of view, the LCA confirmed that mix designs with lower cement content, particularly the 0.15 C/A ratio, consistently minimize environmental impacts across multiple categories, including global warming potential and acidification. By reducing cement content via aggregate optimization, these mixes cut CO_2_ emissions by approximately 30% compared to the reference, demonstrating that strategically lowering cement content is an effective means of curtailing concrete’s overall carbon footprint while maintaining acceptable performance.

## 7. Conclusions

○ This study focused on optimizing concrete designs for minimizing shrinkage, sustaining strength and resistivity, and improving sustainability. By exploring variations in C/A ratios and the incorporation of LWFA by applying the Tarantula curve methodology, insights were gained into the performance of both regular and LWFA concrete mixes. The findings are concluded below:
○ Reducing the C/A ratio in regular concrete significantly improved strength. The 0.165 C/A mix with PLC achieved 42.2 MPa compressive strength (11.9% higher than the 37.7 MPa baseline) and 4.15 MPa flexural strength (48.8% higher than the 2.79 MPa baseline), highlighting the benefits of paste optimization.
○ Lower C/A ratios also reduced shrinkage. The 0.165 mix showed a 12.7% shrinkage reduction (from 355 με to 310 με), enhancing dimensional stability and crack resistance.
○ Decreased paste content led to lower workability (slump dropped from 5.5 cm to 2.9 cm) but increased durability, as seen in higher bulk resistivity (24.5 kΩ·cm at 56 days for C/A = 0.15), indicating improved resistance to ionic transport.
○ Incorporating 15% LWFA in the C/A = 0.15 mix further reduced shrinkage to 265 με (a 19.7% reduction vs. 330 με baseline), showing LWFA’s effectiveness in controlling shrinkage in low-paste mixes.
○ LWFA slightly reduced strength, but the impact was modest. The 15% LWFA mix reached 36.4 MPa in compression (6.3% lower) and 3.57 MPa in flexure (9.3% lower) compared to regular concrete, a worthwhile trade-off for improved shrinkage control.
○ LWFA mixes maintained high bulk resistivity. The 15% LWFA mix recorded 23.7 kΩ·cm, just 3.3% below the regular concrete, suggesting minimal compromise in durability.
○ Lowering cement content, especially at C/A = 0.15, reduced CO_2_ emissions by 30%, underscoring the environmental benefits of material optimization.

## Figures and Tables

**Table 1:** Chemical properties of PLC, Type II cement, and slag.

Oxides	PLC (%)	Type II Cement (%)	Slag (%)
SiO_2_	20.2	21.5	32.70
Al_2_O_3_	3.6	3.7	8.58
Fe_2_O_3_	3.2	3.3	1.70
CaO	65.1	65.2	44.82
MgO	3.0	3.4	9.33
SO_3_	3.4	3.3	1.16
Na_2_O	0.3	--	0.30

**Table 2:** Summarized Tarantula curve compliance criteria.

Criterion	Description
Criterion 1	Aggregate gradation must lie within Tarantula curve boundaries.
Criterion 2	≥15% of total aggregate volume should be coarse sand (#8 to #30 sieve).
Criterion 3	≥15% of total aggregate volume should be fine sand (#30 to #200 sieve).

**Table 3:** Mix design of regular concrete.

Mix Design	C/A Ratio	Cement (kg/m^3^)	Slag (kg/m^3^)	Water (kg/m^3^)	Coarse (kg/m^3^)	Fine (kg/m^3^)	LWFA (kg/m^3^)	HRWR (kg/m^3^)	AE (kg/m^3^)
1	0.21	190	190	145	972	796	0	0.95	0.07
2	0.21	190	190	145	972	796	0	0.95	0.07
3	0.18	159	159	143	972	796	0	1.13	0.07
4	0.165	146	146	131	972	796	0	1.51	0.07
5	0.15	133	133	120	972	796	0	2.27	0.07
6	0.15	133	133	120	884	884	0	2.27	0.07
7	0.21	190	190	145	972	530	265	0.95	0.07
8	0.18	159	159	143	972	530	265	1.14	0.07
9	0.165	146	146	131	972	530	265	1.13	0.07
10	0.15	133	133	120	972	530	265	1.23	0.07
11	0.21	190	190	145	972	619	177	1.17	0.07
12	0.18	268	268	241.5	972	619	177	0.97	0.07
13	0.165	246	246	221.4	972	619	177	1.17	0.07
14	0.15	224	224	201.5	972	619	177	1.30	0.07
15	0.21	320	320	244	972	708	88	0.58	0.07
16	0.18	268	268	241.5	972	708	88	0.85	0.07
17	0.165	246	246	221.4	972	708	88	1.03	0.07
18	0.15	224	224	201.5	972	708	88	1.18	0.07

Mix design 1 uses Type II cement, while PLC is used starting from mix design 2. Three different levels of fine aggregate replacement with LWFA are adopted: 15% in mix designs 7–10, 10% in mix designs 11–14, and 5% in mix designs 15–18.

**Table 4:** Summary of tests performed.

Test Type	Specimen Dimensions	Number per Mix	Ages Tested	Purpose	Standards
Slump	Fresh mix	1	Fresh	Workability evaluation	ASTM C143
Compressive Strength	100 × 200 mm cylinders	3	7, 14, 28 days	Strength development	ASTM C39
Flexural Strength	150 × 150 × 500 mm beams	3	28 days	Bending performance	ASTM C78
Bulk Resistivity	100 × 200 mm cylinders	3	28, 56 days	Durability/resistivity	AASHTO TP 119-21
Shrinkage	76 × 76 × 286 mm prisms	3	Daily for 21 days	Total shrinkage	ASTM C157

**Table 5:** Slump results.

Mix 1	Mix 2	Mix 3	Mix 4	Mix 5	Mix 6
132 mm	139 mm	127 mm	114 mm	73 mm	89 mm

**Table 6:** Comparison between regular and LWFA concrete.

	Compressive Strength (MPa)	Flexural Strength (MPa)	Total Shrinkage (με)	Bulk Resistivity (KOhm-cm)
0% LWFA	37.39 ± 2.87	3.94 ± 0.69	290 ± 12	24.4 ± 1.50
5% LWFA	36.40 ± 2.76	3.76 ± 0.32	285 ± 14	22.1 ± 1.43
10% LWFA	35.41 ± 2.37	3.65 ± 0.30	265 ± 21	22.4 ± 0.55
15% LWFA	35.05 ± 3.19	3.57 ± 0.16	230 ± 13	23.7 ± 1.27

## Data Availability

The original contributions presented in this study are included in the article. Further inquiries can be directed to the corresponding author.

## References

[1] Safiuddin M., Kaish A.A., Woon C.O., Raman S.N. (2018). Early-age cracking in concrete: Causes, consequences, remedial measures, and recommendations. Appl. Sci..

[2] Kovler K., Zhutovsky S. (2006). Overview and future trends of shrinkage research. Mater. Struct..

[3] Bentz D.P., Jensen O.M. (2004). Mitigation strategies for autogenous shrinkage cracking. Cem. Concr. Compos..

[4] Tran N.P., Gunasekara C., Law D.W., Houshyar S., Setunge S., Cwirzen A. (2021). A critical review on drying shrinkage mitigation strategies in cement-based materials. J. Build. Eng..

[5] Ndahirwa D., Zmamou H., Lenormand H., Leblanc N. (2022). The role of supplementary cementitious materials in hydration, durability and shrinkage of cement-based materials, their environmental and economic benefits: A review. Clean. Mater..

[6] Gholami S., Hu J., Kim Y.R., Mamirov M. (2019). Performance of Portland cement-based rapid-patching materials with different cement and accelerator types, and cement contents. Transp. Res. Rec..

[7] Cao Q., Jia J., Zhang L., Ye H., Lv X. (2021). Experimental study of axial compression of reinforced concrete columns made by environment-friendly post-filling coarse aggregate process. Struct. Concr..

[8] Pinto Dabés Guimarães A.C., Nouailletas O., Perlot C., Grégoire D. (2024). Granular Skeleton Optimisation and the Influence of the Cement Paste Content in Bio-Based Oyster Shell Mortar with 100% Aggregate Replacement. Sustainability.

[9] Guan Z., Wang P., Li Y., Li Y., Hu B., Wang Y. (2022). Mesoscale Finite Element Modeling of Mortar under Sulfate Attack. Materials.

[10] Ghoddousi P., Shirzadi Javid A.A., Sobhani J. (2015). A fuzzy system methodology for concrete mixture design considering maximum packing density and minimum cement content. Arab. J. Sci. Eng..

[11] Qin F., Cheng J., Wen H., Liu H. (2018). Numerical simulation analysis of the dynamic mechanical property of concrete based on 3D meso-mechanical model. Fract. Struct. Integr..

[12] Ukala D.C. (2019). Effects of combined aggregate gradation on the compression strength and workability of concrete using fineness modulus. J. Appl. Sci. Environ. Manag..

[13] Ťažký M., Bodnárová L., Ťažká L., Hela R., Meruňka M., Hlaváček P. (2021). The Effect of the Composition of a Concrete Mixture on Its Volume Changes. Materials.

[14] Li C., Wang F., Deng X., Li Y., Zhao S. (2019). Testing and prediction of the strength development of recycled-aggregate concrete with large particle natural aggregate. Materials.

[15] Wang J., Zhang J., Zhang J. (2018). Cement hydration rate of ordinarily and internally cured concretes. J. Adv. Concr. Technol..

[16] Guo L., Wang M., Zhong L., Zhang Y. (2020). Calculation model for the mixing amount of internal curing materials in high-strength concrete based on modified MULTIMOORA. Sci. Eng. Compos. Mater..

[17] Chen H.J., Wu K.C., Tang C.W., Huang C.H. (2018). Engineering properties of self-consolidating lightweight aggregate concrete and its application in prestressed concrete members. Sustainability.

[18] Karim F.R. (2022). Influence of Internal Curing with Lightweight Pumice Fine Aggregate on the Mechanical Properties of Cement Mortars. Construction.

[19] Abdulrasool A.T., Mohammed S.S., Kadhim N.R., Kadhim Y.N. (2022). Effect of Attapulgite as Internal Curing in High-Performance Concrete with Variable Temperature Curing to Enhance Mechanical Properties. IOP Conf. Ser. Earth Environ. Sci..

[20] Abdulrasool A.T., Kadhim N.R., Mohammed S.S., Alher A.A. (2022). The use of ceramics as an internal curing agent in high performance concrete with variable temperature curing to improve mechanical characteristics. IOP Conf. Ser. Earth Environ. Sci..

[21] Zhang J., Han Y., Zhang J. (2016). Evaluation of shrinkage induced cracking in concrete with impact of internal curing and water to cement ratio. J. Adv. Concr. Technol..

[22] Rajamanickam G., Vaiyapuri R. (2016). Self compacting self curing concrete with lightweight aggregates. Građevinar.

[23] Chatale A. (2024). Study on replacement of fine aggregate with light weighted super absorbent material in internal curing concrete. Int. J. Res. Appl. Sci. Eng. Technol..

[24] Lafikes J., Khajehdehi R., Feng M., O’Reilly M., Darwin D. (2018). Internal Curing and Supplementary Cementitious Materials in Bridge Decks.

[25] Rodríguez-Torres S.D., Torres-Castellanos N. (2019). Evaluation of internal curing effects on concrete. Ing. E Investig..

[26] Wang X., Taylor P., Yurdakul E., Wang X. (2018). An innovative approach to concrete mixture proportioning. ACI Mater. J..

[27] Saykat S., Napper C., Abd-Elssamd A., Ma Z.J. (2025). Optimized Aggregate Gradations for Concrete Mixture Designs.

[28] McArtor E. (2021). Reducing Shrinkage Through Admixtures and Aggregate Gradation. Master’s Thesis.

[29] Gursel A.P., Masanet E., Horvath A., Stadel A. (2014). Life-cycle inventory analysis of concrete production: A critical review. Cem. Concr. Compos..

[30] Tang J., Cao J., Luo H., Chen W., Jia Z., Cunha S., Aguiar J. (2024). The effect of demolition concrete waste on the physical, mechanical, and durability characteristics of concrete. Buildings.

[31] Campos H.F., Bellon A.L., Silva E.R.D.L.E., Villatore M. (2021). Eco-efficient concrete, optimized by Alfred’s particle packing model, with partial replacement of Portland cement by stone powder. Rev. IBRACON Estrut. E Mater..

